# Changes in breathing while listening to read speech: the effect of reader and speech mode

**DOI:** 10.3389/fpsyg.2013.00906

**Published:** 2013-12-09

**Authors:** Amélie Rochet-Capellan, Susanne Fuchs

**Affiliations:** ^1^GIPSA-Lab, CNRS et Université Grenoble Alpes, UMR5216Grenoble, France; ^2^Zentrum für Allgemeine SprachwissenschaftBerlin, Germany

**Keywords:** breathing, respiration, speech production, speech perception, adaptation, loudness, speech rate

## Abstract

The current paper extends previous work on breathing during speech perception and provides supplementary material regarding the hypothesis that adaptation of breathing during perception “could be a basis for understanding and imitating actions performed by other people” (Paccalin and Jeannerod, 2000). The experiments were designed to test how the differences in reader breathing due to speaker-specific characteristics, or differences induced by changes in loudness level or speech rate influence the listener breathing. Two readers (a male and a female) were pre-recorded while reading short texts with normal and then loud speech (both readers) or slow speech (female only). These recordings were then played back to 48 female listeners. The movements of the rib cage and abdomen were analyzed for both the readers and the listeners. Breathing profiles were characterized by the movement expansion due to inhalation and the duration of the breathing cycle. We found that both loudness and speech rate affected each reader’s breathing in different ways. Listener breathing was different when listening to the male or the female reader and to the different speech modes. However, differences in listener breathing were not systematically in the same direction as reader differences. The breathing of listeners was strongly sensitive to the order of presentation of speech mode and displayed some adaptation in the time course of the experiment in some conditions. In contrast to specific alignments of breathing previously observed in face-to-face dialog, no clear evidence for a listener–reader alignment in breathing was found in this purely auditory speech perception task. The results and methods are relevant to the question of the involvement of physiological adaptations in speech perception and to the basic mechanisms of listener–speaker coupling.

## INTRODUCTION

At least since Ainsworth (1939), researchers have been trying to understand the adaptation of breathing during the perception of speech. Some of these investigations were motivated by the idea that the listener’s breathing could be sensitive to, or could reflect, some properties of the speaker’s breathing. According to Ainsworth (1939) or Brown (1962) this adaptation phenomenon might be due to empathy. The adaptation of breathing during speech perception has been investigated in different research contexts, such as understanding the sensitivity of breathing with respect to different auditory and visual stimuli (Shea et al., 1987) or with respect to inter-personal coordination in dialog (Warner, 1979; Guaïtella, 1993; McFarland, 2001). Our investigation of breathing adaptation during speech perception is crucial for the following two topics: (1) the involvement of action systems in perception (e.g., Rizzolatti et al., 2001; Arbib, 2010) and more specifically the theories developed to explain the relationship between speech production and speech perception (e.g., Liberman and Mattingly, 1985; Schwartz et al., 2012) and (2) the challenge of explaining inter-personal coordination in joint action (e.g., Obhi and Sebanz, 2011; Di Paolo and De Jaegher, 2012; Chatel-Goldman et al., 2013) and inter-personal alignment occurring in face-to-face communication (e.g., Giles et al., 1991; Pickering and Garrod, 2004; Garrod and Pickering, 2009). At the intersection of these two topics, the study reported in this paper evaluates whether the properties of listener breathing during auditory speech perception could change according to specific changes in reader breathing. Listening to a speaker without seeing him or her is a frequent situation in daily life, for instance, while listening to a radio program with different speakers and different speaking modes. Our study extends previous work on breathing while listening to speech by investigating the effects of the reader and the speaking mode on listener breathing.

Human breathing is a fundamental biological rhythm, which adapts to multiple situations. Breathing is dependent on various physical and mental states and is sensitive to both conscious and unconscious brain controls (Shea, 1996). Breathing has been extensively monitored and analyzed in physiological research. For example, Aleksandrova and Breslav (2009) as well as Shea (1996) provide comprehensive reviews on the control of breathing. Breathing control is commonly split into *vegetative* and *behavioral breathing*. The control of vegetative breathing is a self-sustaining pace maker that regulates the arterial blood gas status. It is located in the brain stem and can work independently of conscious control, as it is the case, for example, in humans under anesthesia. By contrast, behavioral breathing corresponds to explicit voluntary control (e.g., in speaking, singing, yoga) or non-explicit voluntary control (e.g., changes in posture, physical activity, arousal level, mental activity, emotions) that heavily influences vegetative breathing. Different cortical and subcortical networks are involved in the control of behavioral breathing. Shea (1996) suggests that behavioral breathing is learned by means of proprioceptive and auditory feedback and may be motivated by improving the performance of complex tasks. With practice, complex respiratory patterns can be executed more routinely, with less cortical motor planning.

The relationship between these levels of breathing control is still unclear. For speech-related tasks, a continuum has been suggested from vegetative (or quiet breathing) through inner speech or listening during speech to speech breathing (Conrad and Schönle, 1979). This continuum is visible through the progressive decrease of the inhalation duration relative to the exhalation duration, which is maximal in speech production. McFarland (2001) showed that, while listening to speech, inspiratory durations are shorter than in quiet breathing but longer than in speech production. The difference in inspiratory duration between listening and quiet breathing was a global tendency but was not observed for all subjects.

Breathing has also been investigated during purely perceptual speech tasks, when the listener is not speaking. Ainsworth (1939) analyzed the breathing patterns of subjects who did not stutter when listening to subjects who stuttered. Subjects were seated and facing each other. Ainsworth found that breathing when listening to stuttered speech was more variable than breathing when listening to normal speech. This variability decreased over time and was smaller when the listener expected the speaker to produce stuttered speech. Ainsworth suggested that these changes in listener breathing were related to empathy. Brown (1962) assumed that breathing while listening to speech would have different profiles than vegetative breathing. His core hypothesis was that breathing adaptation would occur during speech perception as an empathic response that could actively contribute to speech processing. In particular, he was expecting good listeners to adapt their breathing more to the speaker than poor listeners. Listener breathing was recorded while listening to a female speaker sitting in front of them. The female speaker was always the same and recited a memorized talk about a neutral topic, trying to speak consistently for all the listeners. Brown found that while listening to speech, breathing rate was greater and variability in inhalation depth was smaller as compared to vegetative breathing. However, he did not find clear differences in breathing patterns during listening between good and poor listeners.

Breathing while listening to speech has also been investigated when listening to external stimuli. Shea et al. (1987) systematically tested the impact of visual (three conditions: eyes closed, eyes open, reading a text) and auditory (three conditions: no auditory input, listening to white noise, listening to a recorded text spoken) stimuli on subject breathing. Their main finding was that watching or listening to external stimuli increases the respiratory frequency significantly in comparison to other conditions (no stimulus or neutral stimulus). Breathing frequency with eyes open was different than breathing with eyes closed. No reliable differences were observed for the auditory experiment between no noise and white noise conditions. The results show that breathing is highly sensitive to external stimulation, which calls into question the methods used to record vegetative breathing in studies comparing vegetative and behavioral breathing. The results also suggest specific adaptation of breathing during the processing of verbal material, since reading a text and listening to a recorded text showed the greatest increase in breathing frequency.

Different studies that are not related to speech have investigated breathing behavior while listening or observing the action of another person. Paccalin and Jeannerod (2000) analyzed subject breathing rate when watching an actor performing a weight lifting action in front of them or when watching a movie of an actor running on a treadmill. The study was motivated by the assumption that production is actively involved in perception. The authors were expecting the observer’s breathing to reflect the actor’s breathing. The effort of the actor was varied (increasing weight, accelerating speed of running). The results show that subject breathing frequency partially increased with increasing effort of the actor. These changes were in the same direction as the actor’s breathing and varied with the actor’s effort. No significant changes in heart rate were found. The authors suggested that this partial adaptation of breathing “could be a basis for understanding and imitating actions performed by other people” (p. 194).

Recently, Pellegrini and Ciceri (2012) investigated the perception of breathing and more specifically sensitivity to another person’s breathing. Subjects were listening to the breathing noise related to different activities via earphones. These activities differed in physical effort and mental concentration. One group of subjects was asked to identify the activities by filling out a questionnaire. Another group of subjects was asked to mimic the breathing sounds they listened to. The results show that imitating breathing is easier than identifying breathing carried out in the different activities. Breathing sounds also seem to involve reliable cues about physical effort, but poorer cues about the nature of the activity. Physical effort could be detected more easily than mental effort from breathing noise. The authors suggest “it’s likely that breathing manipulation induces physiological states similar to those of the mimicked activity, inducing closer identification” (p. 22). This conclusion is in agreement with Paccalin and Jeannerod (2000).

Kuroda et al. (2012) investigated the extent to which listeners share breathlessness when facing another person. Subjects were sitting in front of a person who was asked to hold her breath as long as possible after deep inhalation. The results showed breathlessness in observers’ breathing during observation of the person holding her breath, in particular for persons with a high anxiety trait. The authors suggested, “respiration change might contribute to the mechanisms of empathy in humans” (p. 221). This is in agreement with Ainsworth (1939). Boiten (1998) also found that watching short movie clips selected to evoke different emotions induces specific changes in breathing according to the emotion. These changes mainly consisted of a clear distinction in inhalation duration relative to the whole breathing cycle. Inhalation duration was relatively shorter while watching pleasant movies (e.g., funny and tender) in comparison to unpleasant movies (e.g., sad clips and clips showing torture).

This short review shows that breathing during perception differs from vegetative breathing. It appears to be influenced by the action of another person and by perceived emotional states. In particular breathing is highly sensitive to the effort of the perceived action. These influences are visible in global parameters of breathing such as breathing variability, but also in temporal parameters such as the relationship between inhalation and exhalation duration. A detailed analysis of breathing patterns during speech perception is crucial to gain a better understanding of the role of speech production in speech perception. A controlled situation where a group of subjects listens to the same speech material can be taken as a referent/baseline situation. It allows isolating different effects (e.g., speaking mode) and testing specific parameters that might influence listener’s adaptation. As discussed later in the current paper, this approach is complementary to the study of real dialog situations (such as McFarland, 2001).

Stephens et al. (2010) used a so-called “playback” paradigm in order to understand the neurophysiological coupling between listeners and speakers. They recorded a native speaker of English and a native speaker of Russian when telling stories in their native language. The recordings were then played back to native English speakers who did not understand Russian. Listener brain activity was recorded while listening to the pre-recorded stories and compared to the speaker’s brain activity when telling the story. The results showed neural coupling between listeners and speakers whose strength was correlated with comprehension of the speaker’s speech. The authors interpreted the results relative to the two topics mentioned above: the involvement of action in perception and the interactive alignment theory. Even if the situation was not a direct communicative situation, the authors concluded that their findings “indicate that during successful communication, speaker’s and listener’s brains exhibit joint, temporally coupled, response patterns” (p. 14428). It is likely that the purely perceptual paradigm of Stephens et al. (2010) shares basic mechanisms with interactive dialog. In both cases, the listener has to process the speaker’s speech, at least through the auditory channel.

Similarly to Paccalin and Jeannerod (2000) and Stephens et al. (2010), and in line with work that investigated breathing changes during speech perception, we used a playback paradigm to study respiratory adaptation while listening to speech. We used pre-recorded texts read in different conditions that we played back to different groups of listeners. In Experiment 1, we tested the effect of vocal effort (loud vs. normal speech) of the reader on listener breathing. A first attempt was also undertaken to investigate the effect of the reader (male vs. female) on listener breathing. In Experiment 2, we controlled for whether the effects observed in Experiment 1 were due the volume level or to the perception of vocal effort. In Experiment 3, we tested the effect of the female reader’s speech rate (slow vs. normal) on listener breathing. The rationale of our approach was to hold the speech content constant while varying the speaking modes in order to test whether and how listener breathing adapts to these changes. We chose to vary speaker, loudness and rate, as these parameters have been shown to affect speech breathing. For instance, changes in loudness level are associated with highly specific changes in breathing (Winkworth et al., 1994; Huber, 2007); changes in breathing pauses have been found when reading a passage with different speech rates (Grosjean and Collins, 1979); and speech is also influenced by the speaker’s gender or body type (Hoit and Hixon, 1986; Hoit et al., 1989). In addition, we considered the progression of breathing behavior at key moments of the experiment (between the onset and the offset of a condition; between the offset of one condition and the onset of the other condition). We also controlled for the effect of order of presentation of the speech modes on breathing. Finally, we ran simple analyses of the synchronization between listener and reader breathing. Following previous studies of breathing during speech perception and during the perception of action, we hypothesized that differences in reader breathing patterns could induce specific changes in listener breathing.

## MATERIALS AND METHODS

### READERS AND LISTENERS

The readers recorded to build the corpus of read speech used in the listening task were two native speakers of Standard German, a female (age: 35 years old, height: 170 cm, weight: 58 kg) and a male (23 years old, 186 cm, 65 kg). The female reader was selected on the basis of her musical skills. She studied music and sang in a chorus for more than 10 years. The male reader was an athlete exercising at least three times a week. In agreement with the literature of breathing during speech production (Hoit and Hixon, 1986; Hoit et al., 1989) we expected these two persons to show different breathing patterns due to their different morphology, skills, and training.

The listeners were 48 females, all native speakers of German. We choose to involve only female listeners in order to reduce variability in body morphology due to gender and possible complex interactions between listener’s gender and reader’s gender previously described in studies of inter-personal interaction (Bilous and Krauss, 1988). The listeners were selected according to their age and body mass index (BMI), as big variation in weight and height could affect breathing behaviors (Bennett and Zeman, 2004; Parameswaran et al., 2006). Twenty-six listeners were involved in Experiment 1 [age: 25.0 (mean) ± 3.1 (standard deviation); BMI: 21.5 ± 2.1], 10 in Experiment 2 (age: 22.7 ± 4.1; BMI: 21.9 ± 1.7), and 12 in Experiment 3 (age: 27.9 ± 6.2; BMI: 21.3 ± 2.1). None of them reported any history of speech, language, hearing, or breathing difficulties.

### EXPERIMENTAL SET-UP

**FIGURE 1:**
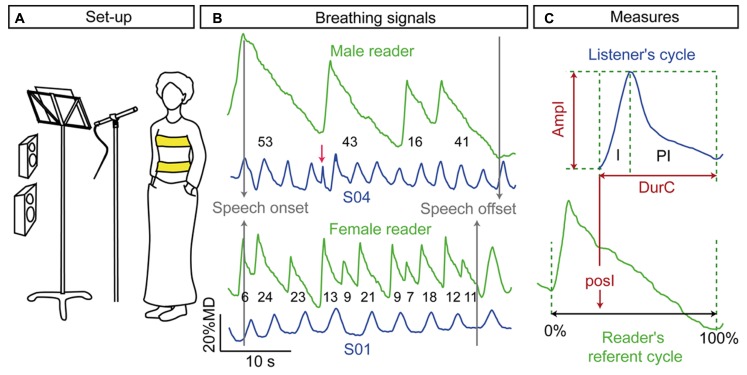
**(A)** Experimental set-up; **(B)** examples of breathing signals produced by the male and the female reader when reading the “The lion and the mouse” with loud speech and examples of listener breathing signals (S01 and S04) while listening to the associated acoustics. The numbers below the readers’ curves indicate the number of syllables. The artifacts such as the peak indicated by the red arrow were discarded; **(C)** the inhalation (*I*) and post-inhalation phase (*PI*) of the breathing cycle were labeled to measure the amplitude (*ampI*) and the duration (*durI*) of the breathing cycle. The onset of listener cycle inhalation was positioned relatively to the closest reader’s cycle, normalized between 0 and 100% (*posI*).

The set-up was the same for the readers and the listeners and is illustrated in **Figure 1A**. Readers and listeners stood in front of a microphone, a music stand (for the speech recording) and two loudspeakers (for the playback paradigm). All participants were asked to maintain their feet aligned with a mark on the floor, to keep their arms along their torso and to try not to move during the recording. The aim of this instruction was to preserve the distance between the speakers and the microphone as well as between the listeners and the loudspeakers, and to minimize body movements that could interfere with monitoring breathing kinematics. The listening task was driven by a computer program on a PC that also played the audio recordings.

### CORPUS FOR THE LISTENING TASK

A few weeks before the experiment, the readers were recorded while reading 20 short texts (fables from Aesop, La Fontaine, and Lessing). In a few cases some words of the original text were changed, since these words are not used any more and sounded antiquated. Readers were instructed to first read all the texts with a normal volume level (*N* condition) and then with a loud volume level (*L* condition). This order was chosen to avoid carryover influences from *L* to *N*. The acoustics and the breathing movements produced during reading were recorded synchronously, as described in Section “Data Acquisition, Processing, and Labeling.” The acoustical signals were processed afterward to select a subset of texts for the listening task. The mean difference in intensity from *N* to *L* was computed for each text and each reader using Praat (Boersma and Weenink, 2011). Fifteen texts were selected and produced with comparable changes in intensity from *N* to *L*. These changes were ~13.1 (mean) ± 2.3 (standard deviation) dB for the male reader and ~12.8 ± 1.5 dB for the female reader. The 15 texts included from 117 to 199 syllables (151 ± 22.1 syllables). Two supplementary texts were chosen as training trials for the listening experiment to introduce the reader’s voice to the listeners in the different conditions. For the purpose of Experiment 3, the female reader was recorded again in a second session, while reading the same texts with a normal volume level but a slow speech rate.

### PROCEDURE OF THE LISTENING TASK

The procedure was the same for the three experiments. Listeners were first recorded while breathing quietly. The recordings of quiet breathing were not further analyzed, since the aim of the study was to compare changes in breathing between different listening conditions. Afterward, listeners were asked to read five texts aloud. They were then instructed that similar texts read by a male or a female reader would be played with different volumes levels (Experiment 1) or different ways of speaking (Experiments 2 and 3). Participants were asked to listen carefully to each text and to shortly summarize its content afterward. This instruction was intended to direct listener attention toward the listening task and toward the content of the text, and away from any focus on their breathing. In the normal condition (*N*), included in all the experiments, each listener heard five texts read with a normal rate and volume, and played back with a normal volume level. They also heard five other texts in a condition that varied according to the experiment, as described subsequently. The order of presentation of the two conditions was counter-balanced across listeners in the three experiments. The texts and their order for each subject were chosen among 15 orders that balanced the number of syllables between conditions. Each condition started with a training trial to familiarize listeners to the reader’s voice for this condition.

In the first experiment, half of the listeners (13 subjects) heard the male reader and the other half heard the female reader. The volume level of the PC and of the loudspeakers were adjusted using a SPL meter such as that the volume heard by the listener (at the position of the microphone) was between 65 and 70 dB in the normal condition, which was equivalent to the readers’ original volume in this condition. Five texts were played in the normal condition described above and five texts were played in the loud condition (*L*). In this latter condition, the texts were read and played with a loud volume level.

In the second experiment, all the listeners heard the female reader only, five texts read with a normal volume level and five texts read with a loud volume level. The normal condition was the same as in Experiment 1 (*N*). In contrast to the first experiment the level of intensity of the five texts in the loud condition was changed to be similar to the normal condition (*Ln* condition). The texts were played back with similar volume level in *N* and *L*.

In the third experiment, listeners also heard the female reader only, five texts in the *N* condition and five texts read with a normal volume level but a slow rate (*S* condition). The volume level of the recording in the *S* condition was adapted to be equivalent to those in *N*. All texts were played back with a normal volume level.

At the end of each experimental session, all the participants (listeners and readers) were asked to do the vital capacity (VC) maneuver that allows evaluating the amplitude of the rib cage and the abdomen movements when subjects go from their lowest lung volume level to the maximal one. To do so, subjects were asked to exhale as much as they could and then to inhale as much as they could. They reproduced the maneuver three times with pauses in-between.

### DATA ACQUISITION, PROCESSING, AND LABELING

#### Recording of acoustic and breathing signals

The acoustic signals were recorded by means of a super cardio condenser microphone (Sennheiser HKH50 P48) coupled with a pre-amplifier. The movement of the rib cage and the abdomen were monitored using an Inductance Plethysmograph (Inductotrace, formerly Respitrace^TM^). The acoustic and the breathing signals were recorded synchronously by means of a six channels voltage data acquisition system (DATaREc^®^ 4DIC6B/DIC6L) and a computer program that sampled acoustic and breathing signals at 11030 Hz. Simultaneous recordings of acoustic and breathing signals were collected during the listening task. This allowed the output of the loudspeakers to be acquired together with listener breathing. One band of the Inductotrace system was positioned at the level of the axilla (rib cage) and the other band at the level of the umbilicus (abdomen; see **Figure 1A**). The circumferences of the two elasticized bands change during inhalation and exhalation and allow estimating the expansion and the compression of the rib cage and of the abdomen during breathing. The gains on the data acquisition unit of the Inductotrace were set-up to be the same for both the rib cage and the abdomen (both values were set to 3). These gains for the rib cage and the abdomen were used for all the subjects involved in the study.

#### Post-processing of the breathing signals

The recordings were post-processed through the following steps: kinematic signals of the rib cage and the abdomen were band-pass filtered at 1–40 Hz (FIR filter) and resampled at 200 Hz. The filtering ameliorated background noise and improved peak detection. The aim of resampling was to reduce the required storage size, as a 200-Hz sampling frequency is sufficient for the reliable detection of the onset and offset of inhalations. During the listening and the reading tasks, we observed variable behavior of abdominal movements among listeners but also for the same listener in different trials. Inhalation and exhalation phases were consistently labeled on the rib cage signal, but not on the abdomen, since its amplitudes were sometimes marginal. We did not want to restrict the analyses to the rib cage movements, since we used a large data sample and some subjects clearly used their abdomen during listening. Moreover, listening to speech could involve the abdomen much more than producing speech. Accordingly we used the sum of the rib cage and abdomen movements to track the onset and the offset of inhalation and to evaluate how the amplitude of inhalation displacements changed from one condition to another. Since our recordings were not calibrated for each subject, the amplitudes we measured are not direct estimations of lung volume. The following paragraph explains this issue in more detail, and the rationale for our final choice to sum the rib cage and abdomen and to analyze both amplitude and duration of the movements.

Since Konno and Mead (1967) it is well known that lung volume can be estimated using an Inductance Plethysmograph from the weighted sum of the rib cage and the abdomen displacements. Usually the relative contribution of the rib cage and the abdomen displacements to the lung volume is computed for each subject using different methods. It has been shown that these methods are more or less reliable (Konno and Mead, 1967; Sackner et al., 1989; Banzett et al., 1995; Barbosa et al., 2012). With the Inductotrace, using a fixed factor of 2:1 (rib cage to abdomen) for all subjects allows a reliable estimation of the lung volume, at least for subjects with standard morphology (Banzett et al., 1995; see also Poole et al., 2000 for an evaluation with children). Banzett et al. (1995) also found that using the same gain for the rib cage and the abdomen does not strongly affect the estimation of lung volume when the subject is in a standing position and does different breathing exercises. According to these authors, the use of the same relative gain for the rib cage and the abdomen for all subjects has the advantage of being easy to reproduce, while subject-specific manipulations of the gain could be inconsistent for the same subject at different moments and/or using different methods. In our data set, the rib cage movements were mostly synchronized with abdominal movements, especially during breathing while listening. Hence the temporal information about the breathing cycle did not depend on the relative gain of the rib cage and abdomen. By contrast, the amplitude of the movement varies according to the weight of the rib cage relative to the abdomen. We first ran all our analyses using an un-weighted sum (1 rib cage + 1 abdomen). In order to be sure that the effects we observed with these signals were not due to an over estimation of the abdomen contribution relative to the thorax, we also analyzed the amplitude using a weighted sum of two rib cage to one abdomen (as suggested by Banzett et al., 1995). Since the conclusions were consistent for both analyses, only the results for the un-weighted sum will be reported here.

In order to compare inhalation depth between subjects, amplitudes have to be expressed in a common scale. When direct airflow measures are available, volume can be expressed in liters. However as all subjects do not have the same lung capacity it seems reasonable to express inhalation depth relative to their maximal capacity as it is commonly reported in the literature. For this purpose, we have normalized the signals by the maximal displacement (MD). The MD was computed for each subject as the maximal change in amplitude among the three VC maneuvers. In the remainder of the paper, the amplitudes of displacement are expressed in percentage of MD (%MD). We did not use percentage of VC (%VC) as in previous work to avoid wrong comparisons, as our recordings were not calibrated.

#### Post-processing of the acoustic signals

The acoustic signals were labeled in Praat. For the readers’ productions, the inter-pausal intervals were labeled and the spoken text produced for each interval was orthographically transcribed. Syllable counts for each breathing cycle were derived automatically from this transcription using the BALLOON toolkit (Reichel, 2012). For the listeners we marked the onset and the offset of the sound recorded from the loudspeaker. This allows detecting the onset and offset of the listening task and to align listeners’ and readers’ breathing movements relative to the onset of the reading. These labels were stored in Praat files (TextGrids).

#### Labeling of the breathing data

The onset and offset of the inhalation phases were automatically detected at 10 and 90% of the velocity peak, respectively. Inhalation is a fast movement and therefore the velocity peak was unambiguous and allowed a reliable detection of the onset and offset of the movement. In contrast, exhalation was much slower, especially during speech production. Its velocity peak was unclear and thus the offset of exhalation was much more difficult to determine. For these reasons, we considered the inhalation phases (*I* on **Figure 1C**) and the post-inhalation phases (*PI* on **Figure 1C**). *PI* corresponded to the phase going from the offset of an inhalation to the onset of the next inhalation. This phase included the exhalation and potential plateau at the end of inhalation and/or exhalation. The labeling was exported to the same file as the acoustical labeling. The breathing signal and the associated labeling were then visualized and the boundaries of the breathing cycles were corrected when required. Artifacts due to non-breathing body movements (see **Figure 1B**) were discarded.

### CALCULATED PARAMETERS AND DATA SELECTION

From the labeling described above, we computed the amplitude (in %MD), and the duration of the breathing cycle (**Figure 1C**). The amplitude (*ampI*) was the displacement from the onset to the offset of inhalation. The duration was the time delay from the onset of one inhalation to the onset of the next inhalation (*durC*). For each listener, breathing cycles with *ampI* and *durC* at more than 1.5 the inter-quartile range from the first and third quartiles were excluded from the analyses. On average this excluded ~10% (~ ±1% standard error) of the breathing cycles during listening in the three experiments. These outliers could correspond to sighs or noises due to body movements.

Finally, we investigated the coordination between reader and listener breathing cycles. To do so, we aligned listener and reader breathing signals relative to the speech onset (see **Figure 1B**). We then positioned each listener inhalation onset with respect to the reader’s corresponding breathing cycle (*posI*, expressed in percent, see **Figure 1C**). For each reader’s breathing cycle, we also computed the syllable rate (*rSyll*), as the number of syllables divided by the duration of the corresponding speech chunks.

### STATISTICAL ANALYSES

We used Linear Mixed Model (lme4 package in R, version 2.15.2) for statistical analyses. We treated Condition, Order, and Reader as fixed factors and Listener and Text as random factors. The *p*-values were calculated using the method of Monte Carlo sampling by Markov chain (*pMCMC* = Monte Carlo Markov chain; see Baayen, 2008). The alpha was set to *pMCMC* < 0.05. Linear mixed models are reliable when the residuals of the model are normally distributed. For each model, the linearity of the residuals was checked using diagnostic tools. When the residuals were not linearly distributed, which was the case for the analyses of the duration of the breathing cycles in Experiments 2 and 3, the values of all subjects and conditions were transformed to a logarithmic scale (Baayen, 2008).

### MAIN QUESTIONS

The data were analyzed with respect to the following main questions: (A) Are listener breathing patterns affected by the Reader (Experiment 1 only)? (B) Are listener breathing patterns sensitive to the Condition and are these effects reflected in the changes observed for the readers? (C) How do listener breathing kinematics adapt to the listening task over time, i.e., from one listening trial to another? (D) Do listener breathing patterns temporally align with the reader breathing? We did not have any specific hypothesis about the Order effect.

## RESULTS

In this section, we first report changes in readers’ breathing behavior according to changes in loudness and speech rate. We then analyze listeners’ breathing kinematics according to the reader they listen to and to the loudness (Experiment 1), according to the vocal effort (Experiment 2), and according to the speech rate (Experiment 3).

### READERS’ BEHAVIOR

In the literature of speech breathing it has been shown that breathing profiles change with gender (Hoit et al., 1989), variations in loudness (Huber, 2007), and speech rate (Grosjean and Collins, 1979). Thus, we were expecting different breathing profiles for the two readers and differences in the way they realized vocal effort and rate.

**FIGURE 2:**
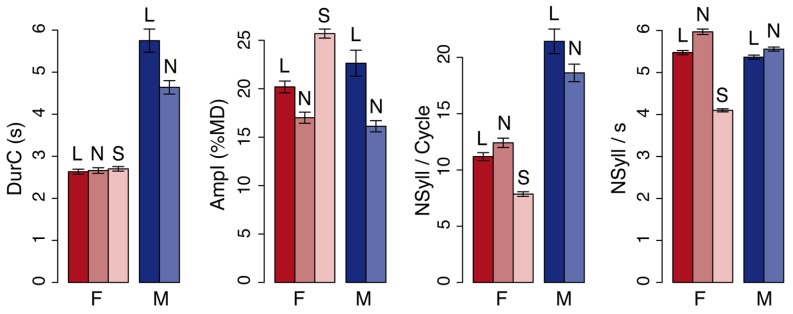
**Readers’ behavior.** From left to right: duration of the breathing cycle, amplitude of inhalation, number of syllables, and syllable rate of the breathing cycle. The values are means and standard errors for all observations. Data are plotted separately for the female (F) and the male reader (M) in the different speaking conditions – loud (L), normal (N), and slow (S). The two readers showed different breathing behavior according to the parameter and the condition (see text for details).

In general, the female reader produced breathing cycles with shorter duration (-2.5 s, Reader: *t* = 16.9, *pMCMC* < 0.001), smaller amplitude (-0.4%MD, Reader: *t* = 2.4, *pMCMC* = 0.02), and fewer syllables (-8.1 syllables, Reader: *t* = 12.1, *pMCMC* < 0.001) than the male reader (see example of a selected breathing signal in **Figure 1B**; average values are given in **Figure 2**).

Changes in breathing behavior due to the loudness level were also reader specific with (a) longer durations in *L* as compared to *N* for the male reader (+1.1 s, Reader × Condition: *t* = -4.5, *pMCMC* < 0.001), but no changes for the female reader; (b) deeper inhalations in *L* as compared to *N* (+4.3%MD, Condition: *t* = -3.5, *pMCMC* = 0.002) for both readers, but with larger changes for the male reader (Reader × Condition: *t* = -2.3, *pMCMC* = 0.02); and (c) a greater number of syllables produced on a single breathing cycle for the male reader in *L* as compared to *N* (+2.8 syllables), while the reverse was observed for the female reader (-1.2 syllables, Reader × Condition: *t* = -3.4, *pMCMC* < 0.001).

The average syllable rate did not significantly change between readers but was greater in *N* than *L* (+0.4 syl/s, Condition: *t* = 7.0, *pMCMC* < 0.001), especially for the female reader (+0.5 syl/s, Reader × Condition: *t* = -2.6, *pMCMC* = 0.009).

When reading slowly (*S*), the female reader produced fewer syllables than in normal condition *N* (-1.9 syl/s, Condition: *t* = -26.7, *pMCMC* < 0.001). She also produced deeper inhalation in *S* as compared to *N* (+8.7%MD, Condition: *t* = 11.8, *pMCMC* < 0.001) and fewer syllables per breathing cycle (-4.5 syllables, Condition: *t* = -10.88, *pMCMC* < 0.0001). The duration of the breathing cycle was similar in the *S* and *N* conditions.

These analyses show that the breathing profiles were different depending on the reader, on the loudness level and on the speech rate, as previously reported in the literature. We then investigated how listener breathing changed according to the reader and to the condition.

### LISTENERS’ BEHAVIOR

Examples of listeners’ breathing while listening to *L* speech produced by the male and the female reader are given in **Figure 1B**. Our main hypothesis was that listener breathing should change in the direction of the reader breathing. To test this hypothesis, we ran a first experiment in which we investigated whether listener breathing kinematics (amplitude of inhalation (*ampI*), and duration of breathing cycle (*durC*)) changes according to the Reader and to the loudness level [Condition: Loud (*L*) vs. Normal (*N*)]. In two supplementary experiments, we tested (Experiment 2) if the effects observed in Experiment 1 were due to the higher volume level of the sound or to the perception of the vocal effort [Condition: Loud played with normal volume (*Ln*) vs. Normal (*N*)] and (Experiment 3) if listeners’ breathing adapts to speech rate [Condition: Slow (*S*) vs. Normal (*N*)].

In the three experiments, subjects listened to five texts in each condition. The order of presentation of the conditions was counter-balanced across listeners and Order was included as a fixed factor in the statistical design [Order: Loud condition first (*LN)* vs. Normal condition first (*NL*) in Experiment 1; Loud played normal first (*LnN*) vs. normal first (*NLn*) in Experiment 2; and Slow played first (*SN*) vs. Normal first (*NS*) in Experiment 3].

#### Experiment 1: the effect of reader and loudness

Experiment 1 involved two groups of 13 female subjects listening to Normal (*N*) and to Loud (*L*) speech produced by the male or the female reader. If listener breathing adapts in the direction of the reader’s breathing behavior we might expect the following: an effect of Reader on *durC* with longer breathing cycles for subjects listening to the male as compared to subjects listening to the female reader (question A); an effect of Condition on *durC* and on *ampI* with larger *ampI* in *L* as compared to *N* for both readers, but longer *durC* in *L* than *N* when listening to the male reader only (B). We also provided information about the questions C and D by testing how adaptation might develop progressively over trials (C) and how listener and reader breathing might be aligned temporally (D).

**FIGURE 3:**
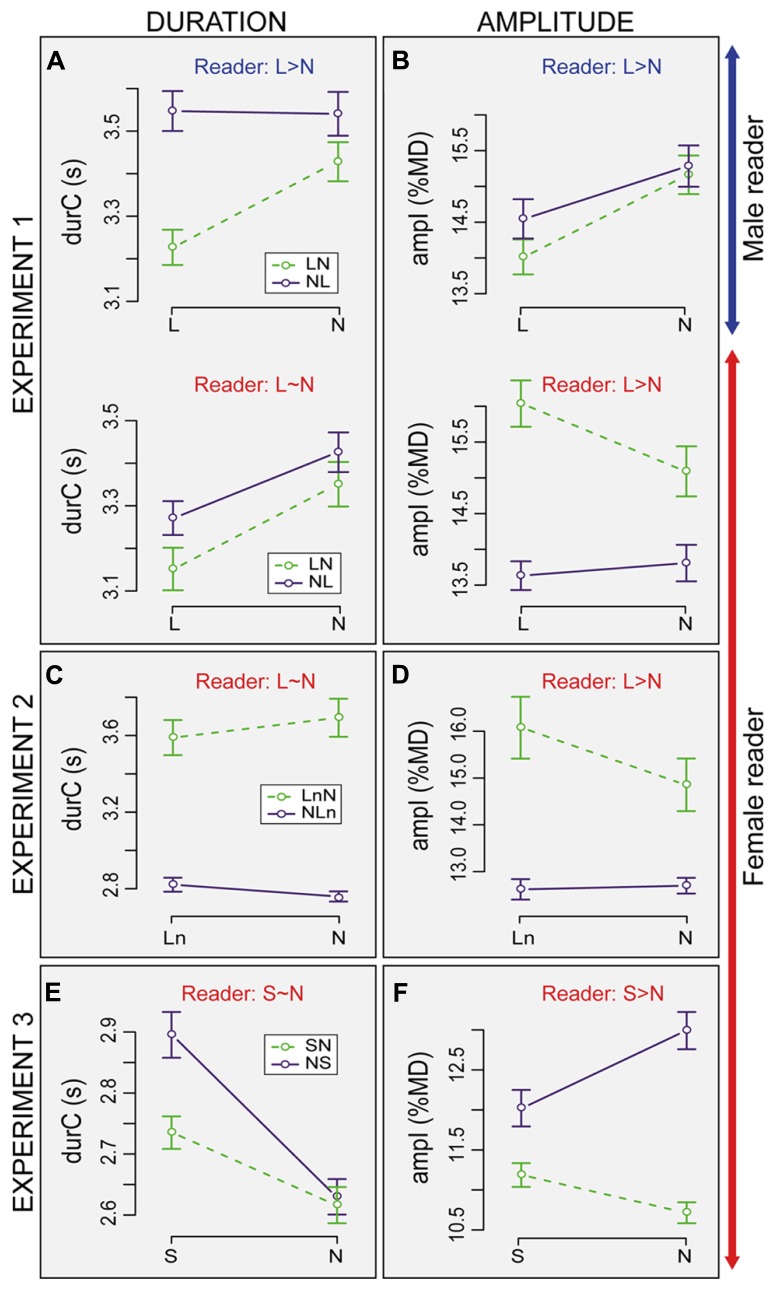
**Average duration (*durC*) and amplitude of inhalation (*ampI*) of listener breathing cycles in the three experiments.** Data are split according to the speech condition (*N*: Normal, *L*: Loud, *Ln* Loud played with normal volume, *S*: Slow) and to the order of presentation (*LN*: loud first; *NL*: normal first, etc.). Bars are ±1 standard error from the mean. The text labels on the top of each graph are a reference and indicate the direction of the effect for the readers for each parameter. Results for duration **(A,C,E)**, amplitude **(B,D,F)**, Experiment 1 **(A,B)**, Experiment 2 **(C,D)**, Experiment 3 **(E,F)**.

***Average changes in listener breathing according to loudness and reader.*** On average, taken all conditions together, listener inhalation depth (*ampI*) was 14.7%MD for subjects listening to the male and to the female reader. The duration of the breathing cycle (*durC*) was 3.3 s for subjects listening to the female and 3.44 s for subjects listening to the male reader. These parameters were sensitive to the Condition in different ways. Interactions were also observed between the different factors (see **Figures 3A, B**).

The average breathing cycle was shorter in *L* as compared to *N* (-139 ms, Condition: *t* = 4.6, *pMCMC* < 0.0001; **Figure 3A**). The main effects of Reader and Order on *durC* were not significant but a three-way interaction showed that the effect of Condition also depended on Reader and Order (Condition × Reader × Order: *t* = -2.1, *pMCMC* = 0.036). When listening to the female reader, *durC* was shorter in *L* than *N* for both orders.** In contrast, when listening to the male reader, the effect of Condition was observed in the *LN* order only. In *NL* order, *durC* was similar in both conditions. The effect of readers was also greater in *L* and *NL* order than in the other conditions (+270 ms for subjects listening to the male).

On average, inhalation amplitude was smaller in *L* as compared to *N* (-0.23%MD, Condition: *t* = 3.1, *pMCMC* = 0.003; **Figure 3B**). As for *durC*, the main effects of Reader and Order on *ampI* were not significant. Yet, subjects listening to the male showed deeper inhalation in *N* than *L* (+0.9%MD) whereas the opposite was observed for the subjects listening to the female reader (-0.47%MD, Condition × Reader: *t* = 6.5, *pMCMC* < 0.0001). These effects also depended on the Order (Condition × Order × Reader: *t* = -4.0, *pMCMC* < 0.0001). The effect of Order was not significant when listening to the male reader. In both *LN* and *NL* orders, *ampI* was deeper in *N* than *L.* In contrast, when listening to the female reader, the inhalation amplitude was larger in *L* than in *N* in the *LN* order. In *NL* order, the difference between *L* and *N* was not significant.

**FIGURE 4:**
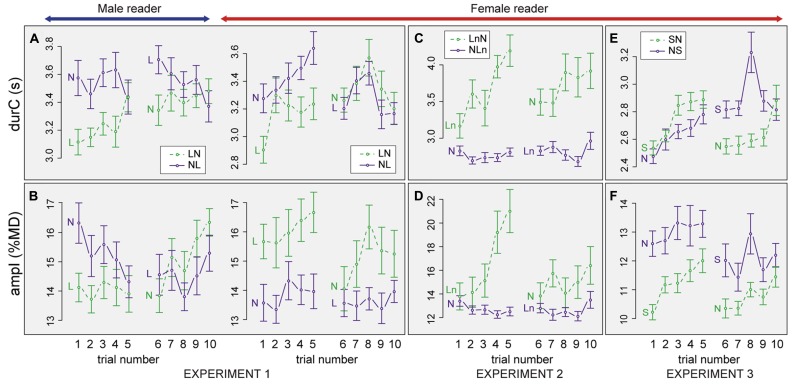
**Progression of listener inhalation amplitude and breathing cycle duration (mean ± 1 standard error) from trial 1 to 10.** Data are split by experiment (Experiments 1, 2, and 3), condition (*N*: Normal, *L*: Loud, *Ln* Loud played with normal volume, *S*: Slow), and Order (e.g., *NL*: Normal-Loud). Results for duration **(A,C,E)**, amplitude **(B,D,F)**, Experiment 1 **(A,B)**, Experiment 2 **(C,D)**, Experiment 3 **(E,F)**.

***Progression of listener breathing over trials.*** In order to better understand the process of listener adaptation, we analyzed the progression of *durC* and *ampI* over the 10 trials. **Figures 4A, B** show the mean values of the two parameters in the different trials split by Reader. We evaluated the adaptation process in the first condition by comparing trial 1 vs. 5; the change from one condition to the other, trial 5 vs. 6; and the adaptation process over the second condition by comparing trial 6 vs. 10. For the statistical analyses, data were split according to Reader and Order. Trial (with levels 1, 5, 6, 10) was taken as fixed factors and Listener as a random factor.

When listening to the male reader in the *NL* order, *durC* was stable for the *N* condition (trials 1–5), did not significantly change when condition changed (trials 5–6) but tended to decrease over the *L* condition (6:10, *t* = -2.1, *pMCMC* = 0.036). *ampI* was decreasing from trial 1 to 5 but the difference was not significant. *ampI* was then stable from trial 5 to 6 and over the *L* condition (trials 6–10). In the *LN* order, *durC* increased over the *L* condition (trials 1:5, *t* = 3.4, *pMCMC* = 0.0008) but was then stable from trial 5 to 6 and over the *N* condition. A reverse tendency was observed for *ampI* that was stable over the *L* condition and when condition changed (trials 1–5, 5–6) but then increased over the *N* condition (trials 6–10, *t* = 3.7, *pMCMC* = 0.0004)

When listening to the female reader in the *NL* order, *durC* progressively increased over the *N* condition (1:5, *t* = 3.0, *pMCMC* = 0.002). *durC* then decreased when condition changed (5:6, *t* = -3.3, *pMCMC* = 0.0008) but was stable over the *L* condition. *ampI* was stable over the ten trials. In the *LN* order, *durC* also increased over trials when listening to *L* speech (1:5, *t* = 4.4, *pMCMC* = 0.0001) but then mainly remained stable from trial 5 to 10. *ampI* did not change significantly over the *L* condition, decreased when condition changed (5:6, *t* = 4.5, *pMCMC* = 0.0002) and increased again over the *N* condition (6:10, *t* = 3.6, *pMCMC* = 0.0006).

In summary, the progression of *durC* and *ampI* over the 10 trials was variable according to Reader and Order. Yet, consistent changes were observed for the two readers in *LN* order for which *durC* increased over the *L* condition and then kept stable, while *ampI* was stable over the *L* condition but then tended to increase over the *N* condition. Changes over trials were less consistent in the *NL* order.

***Temporal alignment of listener breathing to reader breathing.*** The adaptation of listener breathing to the Reader and to the Condition may also occur in the form of a temporal alignment of listener breathing patterns to the reader’s breathing. We analyzed temporal alignment by positioning the onset of each listener’s inhalation relative to the corresponding reader’s breathing cycle (see **Figure 1C**). The value *posI* ranged from 0 to 1, with 0 and 1 corresponding to a synchronization of the listener’s inhalation onset to the readers’ inhalation onset. For example, a value of 0.15 corresponds to the onset of the listeners’ breathing cycle at 15% of the reader’s cycle duration. A value of 0.85 shows that the onset of the listener breathing cycle occurred at 85% within the reader’s cycle.

When two periodic signals are analyzed over time, even when unrelated, some alignment could occur randomly and locally between the two signals. For this reason, we compared the distribution of *posI* for the original listener–reader association with the distribution of *posI* for a random listener–reader association. This comparison between random and real pairs is a common approach in the time-series analyses to test temporal synchronization between two cyclical signals (Lachaux et al., 1999). It was also used in McFarland (2001) using cross-correlations to investigate synchronization during dialog. A random association corresponded to the association of the listener breathing when listening to a given text with the reader breathing when reading another text in the same condition. If listeners temporally align their breathing to the reader breathing we expect: (1) some peaks in the distribution of *posI*; (2) differences between the distributions of *posI* for original vs. random associations.

**FIGURE 5:**
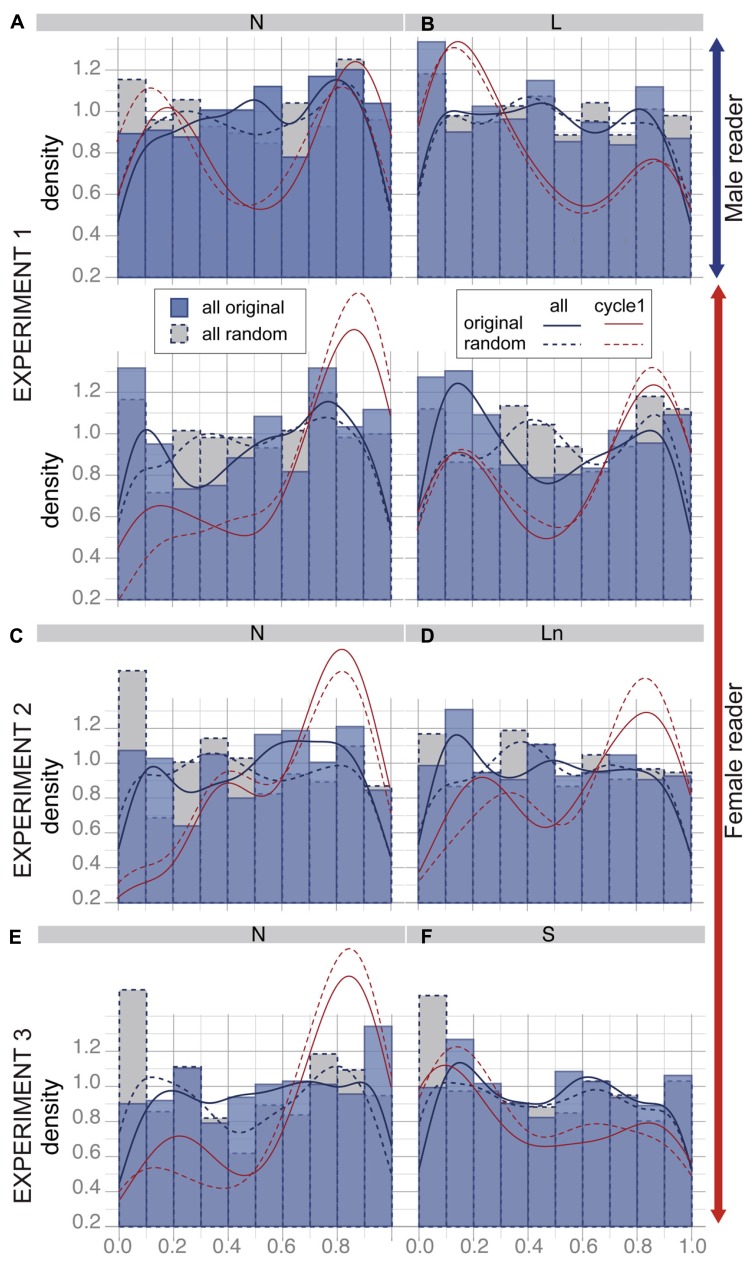
**Distribution of the position of listener inhalation onsets relative to the reader’s breathing cycle (*posI*).** Histograms are distributions of *posI* for the original listener–reader associations (blue) and for corresponding random associations (gray + dotted lines). Curved are density fits corresponding to the distribution of all values (blue) and for the first cycle of each trial (red), for original associations (straight line) and random associations (dotted lines). **A,C,E**: Results for the normal condition in Experiment 1, Experiment 2, and Experiment 3 respectively; **B**: results for Experiment 1, loud speech; **D**: Experiment 2, loud speech normalized in amplitude, **F**: Experiment 3, slow speech.

**Figure 5** shows the distributions of *posI* and their fits for the original associations (blue straight lines) superimposed on the ones for random associations (blue dotted lines). All cycles for all subjects of the respective condition are pooled together. The red curves correspond to the fit of the distributions restricted to the first cycle of each trial. We used Kolmogorov–Smirnov tests to compare the different distributions.

Taking all breathing cycles for subjects listening to the male reader together, the distribution of *posI* does not display any clear peak (see **Figures 5A, B**, first rows). In addition, the distributions of *posI* for the original and the random associations did not differ significantly. This suggests that listener inhalation occurred randomly relative to the male reader breathing cycle. When listening to the female reader, peaks of distribution tend to appear around 0.15 and 0.85, especially when listening to loud speech (see **Figures 5A, B**, second rows). The comparison between distribution of random and original associations tended to be significantly different (*p* = 0.023). This suggests that listener inhalation onset tends to occur close to reader inhalation.

The tendency of listener inhalation onset to occur close to reader inhalation was most frequently found for the first breathing cycle of each trial. In this case, *posI* was mainly around 0.15 and 0.85 for both readers in all conditions. This suggests an alignment between the listener’s first breathing cycle and the reader’s first inhalation. Yet when comparing the distribution of *posI* for the first cycle between original and random associations we did not find any differences. We suppose that this synchronization was probably an artifact due to the onset of the task.

***Summary of the results.*** In summary, listener breathing patterns changed according to the loudness level, but they changed differently with respect to the reader and to the order of presentation. Changes were also found in some cases in the progression of breathing behavior over time. Yet, these changes did not support stable or continuous temporal alignment of listener breathing to reader breathing.

Female subjects listening to the male reader did not have longer breathing cycles than subjects listening to the female reader, except for *L* in *NL* order. The results do not support the expectation (A, see onset of Section “Experiment 1: The Effect of Reader and Loudness”) according to which subjects listening to the male reader should have longer breathing cycles than subjects listening to the female reader. The average ratio between the duration of the breathing cycles for subjects listening to the male relative to the duration of the male reader’s breathing cycle was very small (0.55) for *L* in *NL* (see **Figures 2 and 3**). In other words, in this condition listeners were breathing twice as fast as the reader. This suggests that listeners could produce two breathing cycles for one reader breathing cycle. However, there was no clear temporal alignment between reader and listener breathing cycles.

Subjects listening to the male and the female reader decreased the duration of their breathing cycles when listening to loud speech as compared to normal. Moreover, subjects listening to the male reader decreased the amplitude of their inhalation in loud speech as compared to normal. This was inconsistent with expectation (B) according to which inhalation amplitude should be larger when listening to loud speech as compared to normal. Only the subjects listening to the female reader had deeper inhalation when listening to loud speech in the *LN* order, which is in agreement with expectation (B). Some evidence for progression of adaptation over trials was observed but mainly when loud speech was heard first. In most of the cases an increase in duration and amplitude over trials was found, which partially supports expectation (C) according to which the adaptation could be progressive over time. Finally, no clear temporal alignment occurred between listener and reader breathing, which does not support expectation (D) according to which listener adaptation should temporally synchronize with the reader’s breathing. The differences between subjects listening to the male and the female reader that tended to appear in the loud condition could be explained by the fact that listeners had breathing cycle durations closer to the female reader than to the male, which increases the probability of alignment.

In general, listener breathing kinematics did not clearly mirror reader breathing behavior. Over the trials, the effects of loudness on the duration of the breathing cycle disappeared. When listening to loud speech first, at the end of the five first trials, the durations were similar to those of the group who started with normal speech and similar to the duration of breathing cycles in the subsequent normal speech (five last trials). By contrast, when starting to listen to normal speech, the progression over trials was often not apparent. The effects on duration and amplitude were sometimes inconsistent. This could be due to the fact that the amplitude of inhalation is mainly linked with the duration of inhalation but not necessarily to the duration of whole cycle that includes the *PI* phase (see **Figure 1**).

The changes in breathing due to condition may suggest that listening to loud speech might increase listener breathing rate since it is a stress factor rather than a real adaptation. Therefore, the second experiment was designed to test to what extent the changes observed in Experiment 1 were solely due to the high volume level in the *L* condition, or if they could be linked to the perception of vocal effort involved in the production of loud speech. To avoid gender interaction effects we included only the female reader.

#### Experiment 2: the effect of vocal effort when volume level was unchanged

In the second experiment, the subjects were listening to the female reader only. The listeners were a different group of females (*n* = 10) who did not participate in Experiment 1. The data for the reader were exactly the same as in Experiment 1, but the *L* condition was played back with the same volume level as the *N* condition (it will be now referred to as *Ln* condition). Note that a distinction between the *Ln* and the *N* condition was still audible. If the effects observed in Experiment 1 for the female reader were at least partially due to the perception of the vocal effort and not only the result of a high sound volume level, similar effects of Condition and Order should be observed in Experiment 2. In contrast, if the effects observed in Experiment 1 were a reaction to high sound volume level then no effect of Condition and Order should appear in Experiment 2.

***Average changes in listener breathing according to loudness and reader.*** **Figures 3C, D** summarize the average values of the analyzed parameters (*ampI*, *durC*) according to the factors Condition (*Ln* vs. *N*) and Order (*LnN* vs. *NLn*). Taken all the conditions together, the averages of *ampI* and *durC* were 13.9%MD and 3.15 s, respectively. *DurC* changed with the Order (*t* = -2.2, *pMCMC* = 0.02) and was on average 0.8 s longer in *LnN* than in *NLn*. A main effect of Condition was observed on *ampI* with smaller inhalation amplitude in *N* as compared to *Ln* (-0.5%MD, Condition: *t* = -4.2, *pMCMC* < 0.0001). The effect of Condition on *ampI* also interacts with the effect of Order: changes in *ampI* were observed in the *LnN* order only (+1.2 for *Ln*, Condition × Order: *t* = 3.6, *pMCMC* = 0.0008).

***Progression of listener breathing over trials.*** The analyses of *ampI* and *durC* over trials show different progressions according to the Order (see **Figures 4C, D**). For the *LnN* order, in the *L* condition, both *ampI* and *durC* increased over the five first trials (for *ampI*: 1:5, *t* = 3.1, *pMCMC* = 0.0014 and for *durC*: 1:5, *t* = 3.9, *pMCMC* = 0.0002). Between the fifth and the sixth trial *ampI* and *durC* decreased (5:6, *t* = -3.5, *pMCMC* = 0.0004 for *ampI*, 5:6, *t* = -2.0, *pMCMC* = 0.042 for *durC*), as if listeners were going back to a baseline when the condition changed. Then, they seem to progressively increase *ampI* and *durC* again from the 6th to the 10th trial. Yet, these differences over the second condition showed a trend, but were not significant. By contrast, in the *NLn* order, no clear changes in *durC* and *ampI* were observed over trials.

***Temporal alignment of listener breathing to reader breathing.*** As displayed in **Figures 5C, D** the distribution of the position of the onset of listener inhalation relative to the reader’s breathing cycle (*posI*) were similar to the results of Experiment 1, when subjects listened to the female reader. The main alignment between listener and reader breathing cycles was observed for the first cycle, at the onset of the task and was similar for original and random associations.

***Summary of the results.*** The results in Experiment 2 were in general consistent with the results of Experiment 1 when subjects were listening to the female reader. Listeners show an adaptation in inhalation amplitude toward the female reader who produced deeper inhalations in louder speech. However, this adaptation was only found when listeners heard the speech with more vocal effort first (*LnN* order). Results for duration differ between the two experiments. By contrast with Experiment 1, Condition had no effect on the duration of the breathing cycle in Experiment 2 at a global level. However, as in Experiment 1 in the *Ln* order, duration was increasing from trial 1 to 5. Listener breathing cycles were clearly shorter in the *NLn* group as compared to the *LnN* group. This could suggest unbalanced groups due to small sample size (five subjects in each order). Yet the results for the progression of *ampI* and *durC* over trials show that listeners from the two groups were similar in both duration and amplitude at the onset of the task (for trial 1). The results of this second experiment suggest that results in Experiment 1, when listening to the female reader, might be linked to the perception of changes due to the vocal effort rather than a simple reaction to the sound volume level.

#### Experiment 3: the effect of speech rate on listener breathing behavior

In Experiment 3, we investigated whether changes in speech rate [Slow (*S*) vs. Normal (*N*)] might induce similar effects due to Condition and Order. The aim was mainly to test if listener breathing could reflect changes in the speech rhythm of the reader. This study was run on a new group of female listeners (*n* = 12) and involved the recording of the female reader in normal (*N*) and slow (*S*) conditions. One subject was discarded from the analyses because she showed extremely irregular breathing patterns though the whole course of the experiment. As described in Section “Readers’ Behavior,” the female reader showed similar breathing cycle duration in slow and normal speech. However, she increased the amplitude of the breathing cycle in *S* as compared with *N* and reduced the number of syllables on a single breathing cycle.

***Average changes in listener breathing according to loudness and reader.*** **Figures 3E, F** summarize the average value of the analyzed parameters (*ampI*, *durC*), according to the Condition (*S* vs. *N*) and Order (*SN* vs. *NS*). Taking all conditions together, the average value of *ampI* and *durC* were 12.3%MD and 2.94 s, respectively. *DurC* was in general longer in *S* in comparison to *N* (+178 ms, *t* = 5.9, *pMCMC* < 0.0001). Even if the effect looks greater in *NS* as compared to *SN*, the effect of Order on *durC* was not significant. *ampI* was significantly deeper in *N* than *S* (Condition: *t* = -4.2, *pMCMC* < 0.0001) and deeper in the *NS* than the *SN* order (*t* = -2.3, *pMCMC* = 0.0001). The effect of Condition also changed with Order, with deeper amplitude in *N* than *S* (+1%MD) in the *NS* order, but deeper amplitude in *S* than *N* (+0.5%MD) in the *SN* order (Condition × Order: *t* = 5.6, *pMCMC* < 0.0001).

***Progression of listener breathing over trials.*** Similar to the other experiments, we tested whether listeners changed their breathing behavior over the course of the experiment (Figures 4E,F). For the *NS* order *durC* increased over the five first trials (1:5, *t* = 3.2, *pMCMC* = 0.0026) but then mainly remained stable from trial 5 to 10. *ampI* was relatively stable over the course of the experiment. In contrast, for the *SN* order, *ampI* and *durC* increased from the first to the fifth trial (1:5, *t* = 4.4 and 5.5, *pMCMC* = 0.0001). When condition changed, from *S* to *N*, *ampI* and *durC* decreased (5:6, *t* = -4.0 and -4.7, *pMCMC* = 0.0001). Both values then increased again from the 6th to the 10th trial again (with a tendency for *ampI*, 6:10, *t* = 2.0, *pMCMC* = 0.049 and a significant effect for *durC*, *t* = 3.9, *pMCMC* = 0.0002).

***Temporal alignment of listener breathing to reader breathing.*** The distribution of listener inhalation relative to the reader breathing cycle shows similar profiles as in the two first experiments (see **Figures 5E, F**). Again, no clear temporal alignment was observed, except for the first cycle at the onset of the task. This effect occurred in original and random associations.

***Summary of the results.*** The third experiment suggests that duration of the breathing cycle increased when listening to slow speech as compared to normal speech. This increase did not mirror reader breathing cycle duration since no difference was found between the duration of the breathing cycle in normal and slow speech for the reader. In contrast to reader behavior, listeners decreased the amplitude of inhalation when listening to slow speech as compared to normal speech. Several observations were consistent with the two first experiments. First, at a global level, changes between amplitude and duration according to the condition were not always consistent and the effect of Order was evident. The amplitude of inhalation was deeper when listening to normal speech than slow speech when normal was heard first, while the reverse tended to appear when slow was heard first. Second, the progression over time was less clear when normal speech was heard first. This progression was mainly observed when slow speech was heard first and corresponded to an increase of *ampI* and *durC* over the five first trials. Yet, this increase also occurred over the five trials of the normal speech condition, especially for breathing cycle duration. Third, our analyses of synchronization showed no clear temporal alignment of listener breathing relative to reader breathing except for the first breathing cycle in each trial. This tendency held true in the last experiment, even though the mean ratio between listener and reader breathing cycle duration was close to 1 (see **Figures 2 and 3**).

## DISCUSSION

The aim of this study was to investigate how the breathing of female listeners changes when they listen to a male or female speaker reading short texts with different speaking styles. More specifically, we asked whether a listener breathing during auditory speech perception might change according to specific changes in reader breathing. In general, our results provided evidence for specific changes in listener breathing kinematics dependent on the reader and the vocal effort (Experiments 1 and 2) and to the reader’s speech rate (Experiment 3). Changes were sometimes progressive from the first to the last trial with occasional resetting between conditions. The results, however, did not provide strong evidence that listener breathing consistently reflected or imitated the properties of the reader’s breathing. Partial adaptations in the direction of the reader were mostly found when listeners listened to a reader who was talking in an unexpected speaking style first (loud, with high vocal effort or slow first) and normal second. In addition, listener breathing cycles were not temporally aligned with reader breathing cycles. The results and the methodological limits of the current study are discussed in the context of the literature that has investigated changes in breathing during listening to speech as well as breathing adaptations in direct listener–speaker interactions.

The effect of the reader was investigated in Experiment 1. The results for female listeners’ breathing patterns exhibit some differences with respect to the reader. However, effects were weak and only evident in the interaction between gender and speaking style (loud vs. normal). The interaction effect of reader mainly appeared in the inhalation amplitude, which decreased when listening to the male’s loud speech. It is possible that female listeners could not achieve such deep inhalations, since their lung volume and level of physical training was smaller than that of the male reader. In addition, the synchronization indices were sometimes greater for subjects listening to the female than to the male reader. This last result might be explained by similar body morphology and by the fact that the breathing frequency of female listeners was closer to the breathing frequency of the female reader than the male reader (which also increased the probability of observing synchronization). Complex effects of gender have been observed on inter-personal accommodation in conversation (e.g., Bilous and Krauss, 1988). It may also be possible that phonetic or physiological convergence is not only a property of successful dialog or social factors (Giles et al., 1991) but also a property of similarities in the human brain and body. Such a proposal has been made in the framework of brain-to-brain coupling in human interaction by Hasson et al. (2012), who suggested “If the agent has a similar brain and body, vicarious activations in the perceiver will approximate those of the agent, and the neural responses will become coupled (…). If the agent, however, has a brain and body that are fundamentally different from those of the witness, this vicarious activation pattern will look fundamentally different from that in the agent and the brain responses will not be coupled.” (p. 115). Female listeners in our experiment may be more similar in their brain and body properties to the female reader than to the male reader. It is clear that males and females differ in their body properties, but differences in brain activation during interaction is an open question. Recent brain imaging studies also provide evidence for such differences (see, e.g., Bell et al., 2006).

However, in our study we are unable to generalize and conclude reliably about a gender or reader effect or distinguish between the two, since we only recorded two readers and we also did not test male listeners. Supplementary studies are required to further investigate these effects.

The speech mode of the reader (speaking louder, with more vocal effort or slower than normal) also induced changes in listener breathing. Changing the loudness level was apparently related to perception of the vocal effort, since listening to loud speech played back with normal volume level had comparable effects on listener breathing than listening to loud speech with high volume level. In addition, despite the relatively small number of subjects in each group, the analyses of progression of breathing over time are also consistent between Experiments 1 and 2. This result is in agreement with the adaptation of breathing previously observed during the observation of an action produced with variable efforts (Paccalin and Jeannerod, 2000; see Introduction). Moreover, listener breathing was sensitive to the reader’s speech rate. Breathing cycle duration increased when listening to slow speech as compared to listening to normal speech. However, this adaptation did not reflect changes in the reader breathing as the female reader produced breathing cycles with equivalent duration in the different conditions. Changes in listener breathing behavior could be interpreted as evidence that a slow external rhythm (here the speech rate of the reader) could slow down listener breathing. This result is interesting for applied research, in particular regarding the design of technical systems that could help users to calm their breathing (Wongsuphasawat et al., 2012). The effects of speech rate on the amplitude of inhalation were more difficult to interpret. They also strongly interacted with the order of presentation of the conditions.

Breathing changes were also highly sensitive to the temporal organization of the task. The effect of order suggests that listeners processed the text differently according to the condition. Listeners adapted only when speech was produced with a speaking style different than normal. This speech mode was probably more unexpected or irritating for the listeners who might change progressively to a more relaxed breathing status by increasing the duration of the breathing cycle over time. This result was consistent in all experiments, for *LN*, *LnN*, and *SN* order.

An alternative view is that the effect of order is less an effect due to “surprise,” but a consequence of greater cognitive load (Mitchell et al., 1996) when listeners perceive speech that deviates from normal speech. Cognitive load, however, cannot be linked to the density of purely linguistic information in our study, since readers realized fewer syllables per second in loud or slow speech than in normal speech. Rather, an increased cognitive load may occur when readers produce speech in a style that deviates from normal. Such a view would support the crucial role of “normal” prosody in speech comprehension. Taken together, processing loud or slow speech first in comparison to processing it second (and before getting used to the speaker’s normal speech) might have more emotional implication or generate a higher cognitive load for the listener. Both emotion and cognitive load have been shown to affect breathing during speech perception and production (Boiten et al., 1994; Mitchell et al., 1996).

Due to the small number of trials in this study it is difficult to determine when or whether breathing stabilizes at some point. However, the changes observed over time are consistent with Ainsworth (1939), who found that breathing while listening to stuttered speech changed over time and was less sensitive when listeners were expecting to listen to stuttered speech that normal speech.

Even if listeners do not fully adapt their breathing behavior to the reader, they may show some degree of temporal synchronization. In this study, the main temporal synchronization between listener and reader breathing occurred at the onset of the task, and was probably elicited by the experimenter who started the trial by pressing a key on a keyboard. The listeners most likely took a breath at this moment. We used a simple method to get a first indication of temporal synchronization between listeners and speakers. More elaborate methods that take into account the specific shapes of the listener and reader breathing cycles and the differences in breathing frequencies may provide a better understanding of the temporal organization of synchronization over longer recordings (Pikovsky et al., 2003; Lancia et al., 2012).

It may also be possible that synchronization is limited to certain time intervals or that it requires the listener and speaker to explicitly coordinate their activity, as in the case of turn-taking or topic shift in dialogs (McFarland, 2001). Real dialog situations are the most natural settings to examine listener–speaker mutual breathing adaptation. Few studies have investigated inter-individual adaptation of breathing in face-to-face conversation. Warner (1979) addressed the question of the possible role of breathing rhythm in conversation. In a preliminary study, Guaïtella (1993) showed that speaker breathing adapted to the constraint of the dialog situation and more specifically to turn-taking. McFarland (2001) investigated breathing in face-to-face conversation more systematically, including analyses of coordination using cross-correlations. He found that synchronization between interlocutors breathing occurred mainly at selected intervals where people explicitly had to coordinate, such as turn-taking or when people laughed together. Real dialog includes a number of uncontrolled parameters that restrict the possibilities in testing the roles of specific variables on listener–speaker adaptation. For example, all speakers do not produce the same number of sentences in a dialog and complex inter-relational factors could explain the presence or absence of synchronization as has been shown for phonetic convergence (Bilous and Krauss, 1988; Babel and Bulatov, 2012). For this reason, we believe that the methods used in the current paper are complementary to the analysis of breathing behavior in a real dialog situation. Our experimental design is also similar to a common task such as listening to a radio speaker.

Another important point is the absence of visual cues in our study. We used a playback paradigm similar to Stephens et al. (2010) to investigate reader–listener adaptations. Using such methodology restricts the perception of breathing to auditory cues, while visual cues may be important for inter-personal adaptation of breathing (e.g., Watanabe and Okubo, 1997). Future work should investigate the interaction between auditory and visual cues in more detail, and especially the role of breathing noises in listener–speaker adaptation.

As mentioned in the introduction, this work has ramifications for two main areas: the role of action in perception and the alignment in joint actions. Different studies show a strong relation between the production and the perception of speech. For instance, tongue muscles may be activated during the perception of sounds in which the tongue is involved (Fadiga et al., 2002). In a preliminary study, Weirich and Simpson (2012) have shown that listeners execute vertical tongue movements while listening to speech, although these movements disappear over time (with repetitions). The mutual influences of speech production and perception may extend to the dyad in dialog situations. The speaker’s productions mutually influence the listener’s productions reciprocally through the turn-taking process. The communicative process may progressively lead to linguistic alignment between the two communication partners at different levels (e.g., at the phonetic, phonological, and syntactic levels, see Pickering and Garrod, 2004). Alignment can also occur at the motoric level (e.g., in breathing; Müller and Lindenberger, 2011), posture (Shockley et al., 2003) or in heart rate dynamics (Konvalinka et al., 2011). Hence, the empathic adaptation of breathing during speech perception as suggested by Ainsworth (1939) or Brown (1962) might result from activation of a listener’s speech motor system by the speaker as suggested by theories formalizing the role of speech production in speech perception (Liberman and Mattingly, 1985; Schwartz et al., 2012). In such theoretical frameworks one would expect listener breathing to reflect properties of speaker breathing. If perception is linked to a full activation of the motor system, breathing in speech perception should be similar to breathing in associated speech production. However, such patterns were clearly not observed in our studies. This suggests that the motor activation during speech perception linked with articulatory movements might not propagate to lower levels of control such as breathing. Changes in breathing during speech perception might rather be the product of changes in emotional or cognitive states induced by the processing of external stimuli (Shea et al., 1987; Mitchell et al., 1996).

More studies are required to fully understand how breathing is involved in listener–speaker adaptation using larger populations and longer time windows. We suggest that investigating breathing patterns in listener–speaker interactions will provide new insights into the way physiology is integral to human interaction. Breathing can also be seen as a behavioral window revealing cognitive and emotional processes that potentially allows online tracking of these processes.

## Conflict of Interest Statement

The authors declare that the research was conducted in the absence of any commercial or financial relationships that could be construed as a potential conflict of interest.
